# Virucidal efficacy of guanidine-free inactivants and rapid test buffers against SARS-CoV-2

**DOI:** 10.1038/s41598-021-02942-4

**Published:** 2021-12-03

**Authors:** Katherine Davies, Ulrike Arnold, Hubert Buczkowski, Christopher Burton, Stephen R. Welch, Nicole Green, Rhea Strachan, Tracy Beetar-King, Peter Spencer, Nipunadi Hettiarachchi, Matthew J. Hannah, Matthew Jones, Patricia A. Cane, Christine B. Bruce, Neil Woodford, Allen D. G. Roberts, Marian J. Killip

**Affiliations:** 1 High Containment Microbiology, NIS Laboratories, National Infection Service, Public Health England, London, NW9 5EQ UK; 2 NIS Laboratories, National Infection Service, Public Health England, London, NW9 5EQ UK

**Keywords:** Infectious diseases, Clinical microbiology, Virology

## Abstract

A pathogen inactivation step during collection or processing of clinical samples has the potential to reduce infectious risks associated with diagnostic procedures. It is essential that these inactivation methods are demonstrated to be effective, particularly for non-traditional inactivation reagents or for commercial products where the chemical composition is undisclosed. This study assessed inactivation effectiveness of twenty-four next-generation (guanidine-free) nucleic acid extraction lysis buffers and twelve rapid antigen test buffers against SARS-CoV-2, the causative agent of COVID-19. These data have significant safety implications for SARS-CoV-2 diagnostic testing and support the design and evidence-based risk assessment of these procedures.

## Introduction

The international response to the COVID-19 pandemic has required diagnostic testing of a vast number of patient samples worldwide for the detection of SARS-CoV-2^1^. SARS-CoV-2 is classified as a Hazard Group 3 (HG3) pathogen in the UK by the Advisory Committee for Dangerous Pathogens due to its potential to cause severe respiratory disease; propagative work for any purpose must be carried out at containment level 3 (CL3)^2^, which can severely restrict testing capabilities. Due to the exceptional circumstances presented by COVID-19, non-propagative diagnostic testing may be carried out at a lower level of containment with heightened control measures and suitable, sufficient risk assessment of procedures^2,3^. In the UK, infectious material must be inactivated by a validated method before being handled outside a microbiological safety cabinet (MSC)^2^; selected inactivation methods must be both effective at reducing pathogen infectivity and compatible with downstream sample processing.

Development of rapid tests designed for near-patient or point-of-care (POC) use, including rapid RT-PCR, loop-mediated isothermal amplification (LAMP) and lateral flow immunoassay (LFIA) antigen tests, has permitted SARS-CoV-2 testing to move outside traditional laboratory settings into the community. As for all diagnostic procedures, performing these tests at POC or near-POC requires a suitable and sufficient risk assessment and implementation of appropriate control measures^2–4^: it is essential that the rapid testing procedure, which may involve swab stirring/mixing into an elution buffer with the potential for associated generation of aerosols, does not place individuals performing these tests at an increased risk of SARS-CoV-2 exposure. It is therefore highly desirable that rapid testing procedures include a step that reduces the titre of infectious virus present e.g. by eluting samples into an effective lysis buffer.

The chaotropic agents guanidine thiocyanate (GTC) and guanidine hydrochloride (GHCl) are commonly used to inactivate viruses prior to nucleic acid testing^5^ and guanidine-based lysis buffers are effective at reducing SARS-CoV-2 titre^6,7^. However, reagents containing GTC and GHCl are a chemical hazard in testing laboratories due to their toxicity and incompatibility with sodium hypochlorite-based disinfectants^8^. Their hazardous nature also limits use of GTC and GHCl buffers in home-sampling kits because of the potential for misuse by members of the public. There are now many commercial non-hazardous alternatives to guanidine-based lysis buffers; these would prove valuable additions to COVID-19 testing procedures if effective at inactivating SARS-CoV-2. Currently, there is limited information available on the efficacy of SARS-CoV-2 inactivation by either guanidine-free lysis buffers or by rapid test buffers. This study provides data on inactivation of SARS-CoV-2 by twenty-four guanidine-free molecular extraction reagents and specimen transport media, and twelve LFIA antigen test buffers.

## Methods

All handling of infectious SARS-CoV-2 was performed inside Class III MSCs within a CL3 facility. SARS-CoV-2 (England/02/2020) was propagated in Vero E6 cells (Vero C1008; ATCC CRL-1586) as previously described^6^. Virus was used in inactivation tests at passage 2 or 3 (with virus titres of approximately 1 × 10^7^ PFU per ml). Approximately tenfold concentrated SARS-CoV-2 virus stocks (approximately 1 × 10^8^ PFU per ml) were produced by centrifuging SARS-CoV-2 supernatant through Amicon Ultra-15 (100 K) filters (Millipore) according to the manufacturer’s instructions.

**Figure 1:**
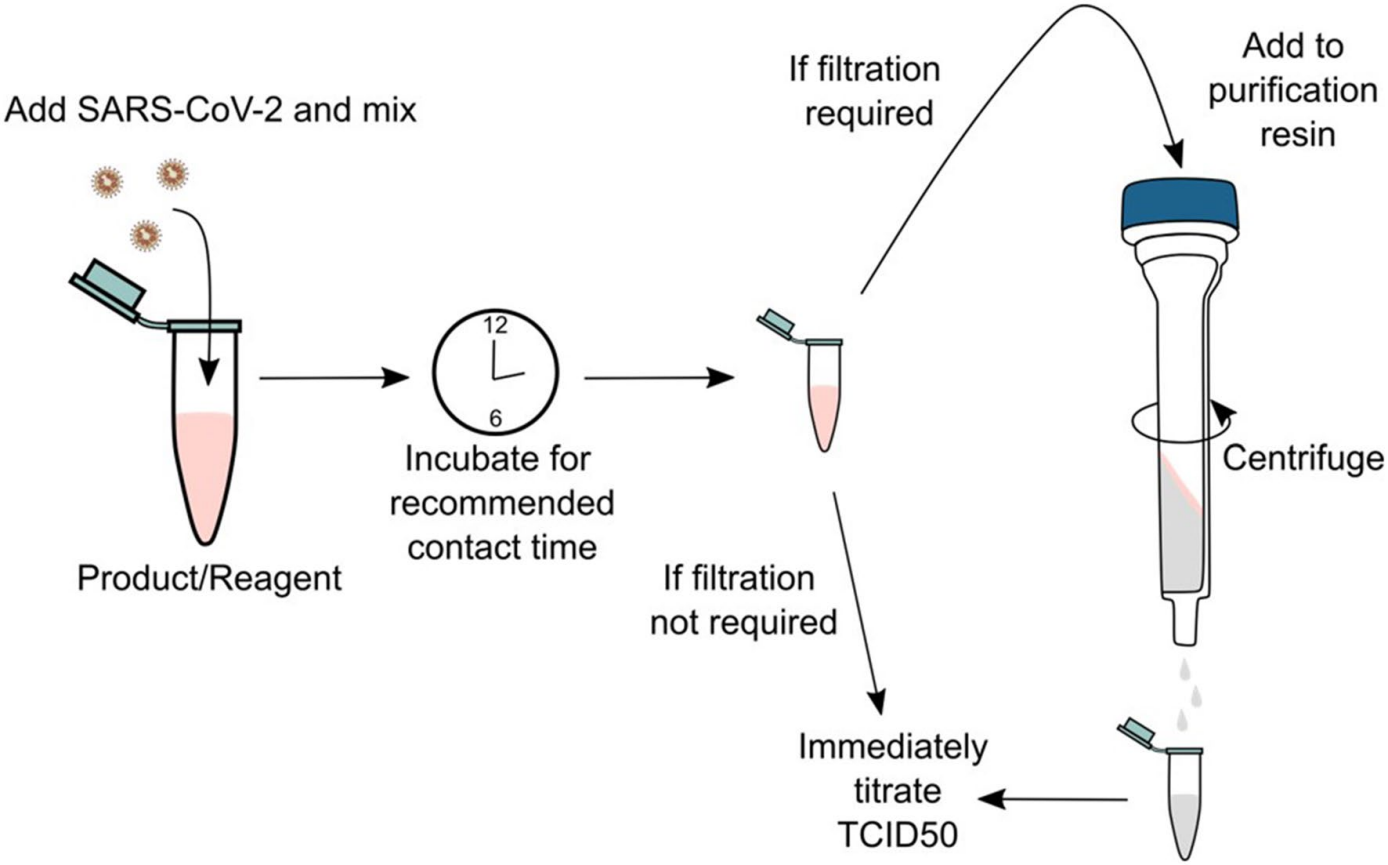
Inactivation testing workflow. SARS-CoV-2 suspensions are treated with guanidine-free molecular extraction reagents or LFIA buffers and mixed before incubating for the recommended contact time. If filtration is required to remove product-associated cytotoxicity, samples are added to a purification resin and centrifuged to elute and immediately titrated. If filtration steps are not required, samples are immediately titrated. Image created in Inkscape version 1.0 https://inkscape.org.

Guanidine-free lysis buffers and LFIA tests kit buffers evaluated and the active ingredients of these products where disclosed are listed in Table 1. Inactivation testing was performed essentially as previously described^6^ and is depicted in Fig. 1. In brief, SARS-CoV-2 was incubated with nucleic acid extraction buffers at a volume ratio and for a contact time indicated in the manufacturer’s instructions, where these were available. LFIA buffers were tested after a 1-min treatment time, to mimic likely contact times in real-life testing scenarios. 5- and 10-min treatment times were additionally evaluated to determine the effect of longer incubation times. All tests were conducted at ambient room temperature (18–22 °C). For most products, treated samples were passed through a filtration matrix (Detergent Removal Resin [Pierce], Sephadex LH-20 [GE Healthcare], Sephacryl S-400HR [Sigma-Aldrich] or BioBeads SM2 [Biorad]) to remove cytotoxic chemicals prior to virus titration to improve the limit of detection (LOD)^6^. The optimum purification method for each product was assessed as previously described^6^, and is listed in Table 1. All experiments were carried out as three independent inactivation tests. After sample clean-up, samples were tenfold serially diluted in PBS and plated on to 96-well plates containing 2.5 × 10^4^ Vero E6 cells. Plates were incubated for 6–7 days at 37 °C/5%CO_2_, then fixed and stained with crystal violet to visualise cytopathic effect (CPE). The 50% tissue culture infectious dose (TCID_50_) was calculated according to the Spearman-Karber method^9^. As a control for virus recovery, SARS-CoV-2 was mock-treated with PBS in parallel, at the same ratio as the reagent being tested. PBS-treated samples were subjected to the same filtration and titration methods as product-treated samples. Titre reduction was calculated by subtracting the mean log_10_ titre of tested samples from the mean log_10_ titre of PBS controls. A cytotoxicity control was purified (if applicable) and titrated alongside other test samples, and used to determine the LOD for each test; this control consisted of PBS in place of virus, treated with an equivalent volume of product as test samples. 95% confidence intervals for titre reductions were calculated as mean log_10_ titre reduction ± 1.96 standard error^9^. Products were tested against an unconcentrated SARS-CoV-2 stock as standard. Concentrated virus preparations were used to increase the maximum possible titre reduction for products (indicated in Table 1) that displayed high levels of cytotoxicity and/or that were previously found to reduce the titre of unconcentrated virus to below the test LOD.

**Table 1:** SARS-CoV-2 inactivation by guanidine-free nucleic acid extraction reagents.

Product name (manufacturer)	Active ingredient/s, if known*	Purification resin^‡^	Reagent: virus ratio	Contact time (min)	Titre reduction, log_10_ TCID_50_/ml [± 95% CI]^†^	Virus detectable in TCID_50_ (virus detected in n/N replicates)
virusPHIX + (RNAssist)	20–90% trifluoroacetamide 20–90% trimethylglycine	PDRR	3:1	10	≥ 6.4 [6.2–6.7]	No^3^
				30	≥ 7.1 [6.9–2.4]	No^3^
virusPHIX-LV (RNAssist)	20–50% trifluoroacetamide 20–50% trimethylglycine	PDRR	10:1^#^	10	≥ 5.1 [4.8–5.5]	No^4^
				30	≥ 6.0 [5.7–6.3]	No
virusPHIX-P9 (RNAssist)	2–5% trifluoroacetamide 1–3% trimethylglycine	LH20	3:1	10	≥ 4.4 [4.1–4.6]	Yes (1/3)
				30	≥ 4.8 [4.5–5.1]	No^3^
virusPHIX-CU (RNAssist)	Urea, choline chloride	None	3:1	10	0.6 [0.1–1.0]	Yes (3/3)
				30	1.0 [0.6–1.4]	Yes (3/3)
VitaPCR SARS-CoV-2 sample collection buffer (Credo Diagnostics)	0.2% sodium hydroxide	PDRR	10:1^#^	1	≥ 5.0 [4.7–5.3]	Yes (3/3)
				5	6.2 [5.9–6.5]	Yes (3/3)
MELT medium B (Mast Group)	1–10% Triton X-100	PDRR	10:1^#^	5	≥ 7.0 [6.6–7.3]	Yes (1/3)
MELT V1 (Mast Group)	Ethylene oxide propylene oxide copolymer mono (nonylphenyl) ether	PDRR	10:1	10	1.4 [1.0–1.8]	Yes (3/3)^1^
				30	1.9 [1.5–2.2]	Yes (3/3)
MELT V3 (Mast Group)	Triton X-100 reduced	SM2	10:1	15	≥ 5.5 [5.2–5.8]	No^1^
MELT V4 (Mast Group)	Triton CG-110	SM2	10:1	15	≥ 5.5 [5.2–5.8]	No^1^
Virus preservation solution (D-Biotech)	Citric acid < 0.1% Triton X-100	PDRR	10:1^#^	10	≥ 6.0 [5.7–6.3]	Yes (2/3)
				30	≥ 5.3 [5.0–5.6]	No^2^
ID NOW COVID-19 elution buffer (Abbott)	0.1% Triton X-100	PDRR	25:1^#^	1	3.6 [3.2–4.0]	Yes (3/3)
				5	4.0 [3.6–4.4]	Yes (3/3)
OMNIgene oral (DNA Genotek)	1–5% SDS	S400HR	1:1	10	≥ 4.3 [4.0–4.6]	No^1^
COVID-19 PROmate sample preparation buffer with standard Triton X-100 (Novacyt)	0.5% Triton X-100	SM2	10:1^#^	2	5.3 [5.0–5.7]	Yes (3/3)
				5	5.9 [5.6–6.2]	Yes (3/3)
				10	≥ 6.2 [5.9–6.6]	Yes (1/3)
COVID-19 PROmate sample preparation buffer with Triton X-100 reduced (Novacyt)	0.5% Triton X-100 reduced	SM2	10:1^#^	2	≥ 6.2 [5.9–6.6]	Yes (2/3)
Saliva RNA sample collector kit (Zeesan)	5–15% sodium lauroyl sarcosinate	PDRR	2:1	30	≥ 5.6 [5.3–5.9]	Yes (2/3)
Virus RNA collection Kit (Zeesan)	5–10% sodium lauroyl sarcosinate	SM2	2:1	10	3.3 [2.9–3.7]	Yes (3/3)
				30	4.5 [4.3–4.8]	Yes (3/3)
Salicovgel-1 (LGC)	Enzymatic	S400HR	10:1	60	1.0 [0.6–1.4]	Yes (3/3)
Salicovgel-2 (LGC)	Enzymatic	S400 HR	10:1	60	3.1 [2.9–3.4]	Yes (3/3)
				180	≥ 3.4 [3.2–3.7]	Yes (2/3)
GeneFix Saliva RNA collector (IsoHelix)	Not available	S400HR	1:1	10	≥ 5.3 [5.0–5.6]	No^4^
BuccalFix (IsoHelix)	Not available	S400HR	5:1^#^	10	≥ 4.7 [4.4–5.0]	No^4^
MicroLYSIS-RNA (Clent Life Science)	Not available	S400HR	1:1	5	1.0 [0.6–1.4]	Yes (3/3)
				20	1.9 [1.6–2.3]	Yes (3/3)
Coronavirus extraction reagent 2506 (Arcis Biotechnology)	Not available	None	2:1	10	≥ 3.3 [3.0–3.6]	No^5^
FRANKD buffer (GeneMe)	Not available	PDRR	10:1	1	3.6 [3.3–3.9]	Yes (3/3)
				5	4.0 [3.7–4.3]	Yes (3/3)
SAVD buffer (GeneMe)	Not available	PDRR	4:1	1	≥ 4.5 [4.2–4.8]	No^5^

See overleaf for table footnotes.

Where identical results were obtained at multiple contact times, only the shortest contact time is shown.

*As indicated on product safety data sheets or other product literature.

^†^Values are given as ≥ when at least one replicate was below the limit of detection.

^#^Tested using concentrated virus stock.

^‡^PDRR: Pierce Detergent Removal Resin; SM2: Biorad SM2 BioBeads; S400HR: Sephacryl S-400HR; LH20: Sephadex LH-20.

*STM* specimen transport media.

Limit of detection: ^1^0.7 log_10_ TCID_50_/mL; ^2^0.8 log_10_ TCID_50_/mL; ^3^1.0 log_10_ TCID_50_/mL; ^4^2.0 log_10_ TCID_50_/mL; ^5^1.7 log_10_ TCID_50_/mL.

## Results

**Figure 2:**
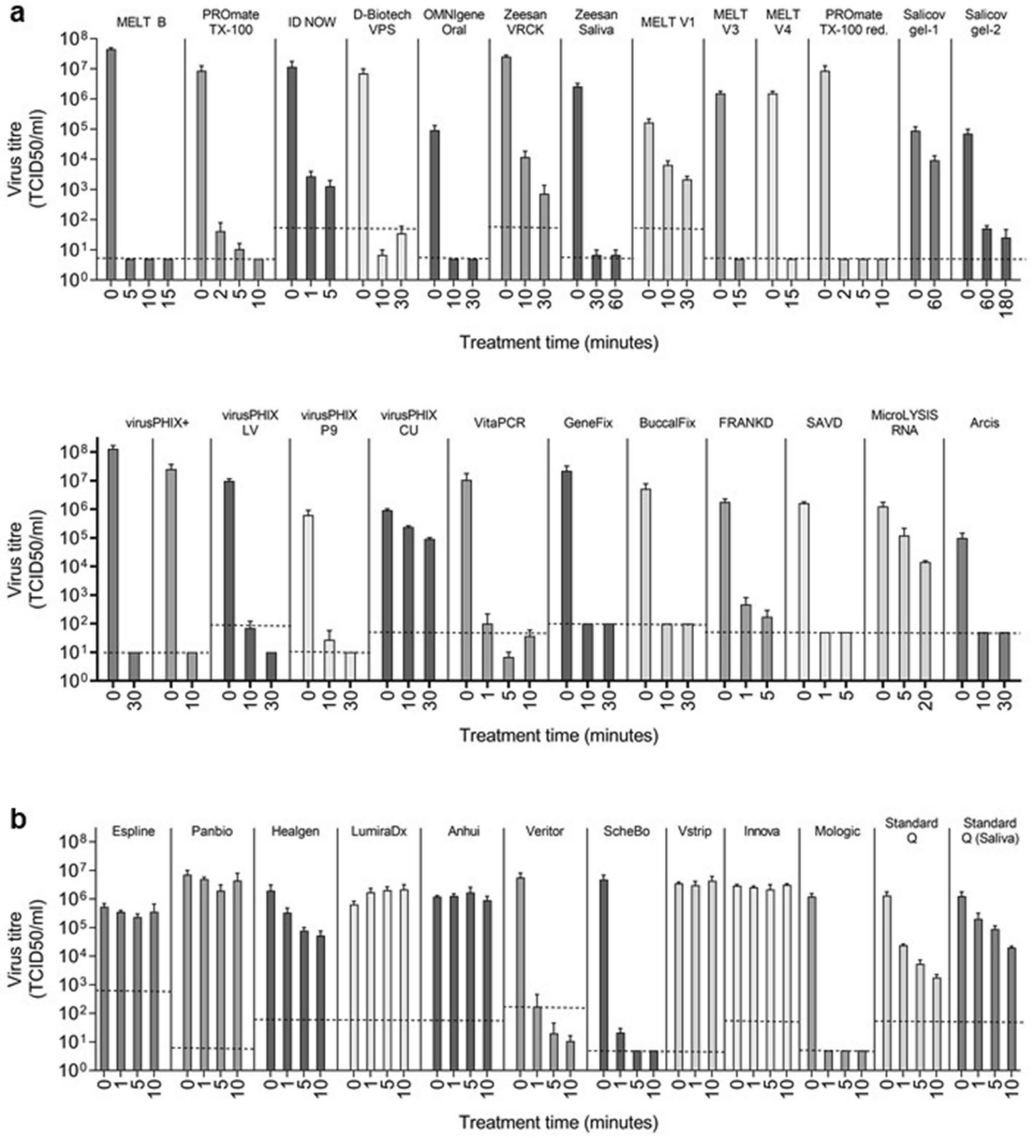
SARS-CoV-2 titre reductions following treatment with guanidine-free molecular extraction reagents and LFIA buffers. SARS-CoV-2 suspension was treated with guanidine-free molecular extraction reagents (**a**) or LFIA buffers (**b**), using the contact times and concentrations stated in Tables 1 and 2, respectively. SARS-CoV-2 was mock-treated with an equivalent volume of PBS in parallel. Samples were purified by methods indicated in Tables 1 and 2 to remove product-associated cytotoxicity, then titrated by TCID_50_ on Vero E6 cells to determine virus titre. All treatments and mock-treatments were performed in triplicate; bars show the mean of triplicate inactivation tests and error bars the standard deviation. The limit of detection for each test was determined using the cytotoxicity control for each test, and is indicated on the graph for each test by a dashed line. Where cytotoxicity of sample replicates and/or treatment times for a product differed, the highest LOD for the entire test is displayed. Variation in virus titres for PBS-treated samples between product tests was due to differences in the titre of virus stock used, the ratio of sample to product or PBS used for treatment, and differing virus recovery following sample filtration with different methods^6^. VRCK: Virus RNA Collection Kit; VPS: Virus Preservation System; Triton X-100 red.: Triton X-100 reduced. Graphs created in GraphPad Prism version 9 https://graphpad.com.

The guanidine-free lysis buffers listed in Table 1 were tested for their ability to inactivate SARS-CoV-2 at contact times determined according the manufacturer’s instructions where available. These reagents are variously marketed as appropriate for lysis of different sample types, including nasopharyngeal/oropharyngeal swabs, sputum and saliva, prior to nucleic acid extraction or as inactivating specimen transport media. A > 4.0 log_10_ reduction in SARS-CoV-2 titre, required by the British European standard BS EN 14476 for quantitative virucidal suspension tests^9^, was demonstrated for most guanidine-free nucleic acid extraction buffers tested (Table 1; Fig. 2a), including: virusPHIX+, virusPHIX-LV and virusPHIX-P9; VitaPCR Sample Collection Buffer; MELT Medium B, V3 and V4; D-Biotech Virus Preservation Solution; ID NOW COVID-19 Elution Buffer (after a 5-min treatment); OMNIgene Oral; PROmate Sample Preparation Buffer containing either Triton X-100 or Triton X-100 reduced; Zeesan Saliva RNA Sample Collector Kit buffer; Zeesan Viral RNA Collection Kit buffer (after 30 min); IsoHelix GeneFix and Buccalfix buffers; GeneMe FRANKD (after 5 min) and SAVD buffers.

Of the molecular extraction reagents tested, we failed to demonstrate > 4.0 log_10_ reduction in SARS-CoV-2 titre for Salicovgel-1 (1.0 log_10_ after 1 h), Salicovgel-2 (3.1 and ≥ 3.4 log_10_ reduction after 1 and 3 h, respectively), MicroLYSIS-RNA (1.0 log_10_ reduction after the recommended 5 min contact time and 1.9 log_10_ reduction after 20 min), virusPHIX-CU (1.0 log_10_ reduction after 30 min) and Arcis Coronavirus Extraction Reagent 2506 (≥ 3.3 log_10_ reduction after 10 min) (Table 1; Fig. 2a).

Only three of the twelve LFIA buffers tested reduced SARS-CoV-2 titre by greater than 4 log_10_ (Table 2; Fig. 2b): the BD Veritor Extraction Reagent (≥ 4.5 log_10_ reduction after a 1-min treatment); the ScheBo SARS-CoV-2 Quick Antigen extraction buffer (5.3 log_10_ and ≥ 6.0 log_10_ reduction after 1 min and 5 min, respectively); and the Mologic Rapid Antigen Test Sample Buffer (≥ 5.4 log_10_ reduction after 1 min). Several LFIA buffers reduced SARS-CoV-2 titre more modestly. Standard Q Extraction Buffer from the SD Biosensor COVID-19 Ag LFIA gave a reduction of 2.9 log_10_ after 10 min. Maximum demonstrable titre reductions for Healgen and Standard Q Saliva LFIA buffers were 1.6 log_10_ and 1.8 log_10_, respectively, after 10 min. ESPLINE, Panbio, LumiraDx, Anhui Deepblue, Innova and Vstrip LFIA buffers had a negligible effect on infectious SARS-CoV-2 , even after a 10-min contact time (Table 2; Fig. 2b).

**Table 2:** SARS-CoV-2 inactivation by rapid antigen test buffers.

Product name (manufacturer)	Active ingredient/s, if known*	Purification resin^‡^	Reagent: virus ratio	Contact time (min)	Titre reduction, log_10_ TCID_50_/ml [± 95% CI)^†^	Virus detectable in TCID_50_ (virus detected in n/N replicates)
ESPLINE SARS-CoV-2 extraction solution (Fujirebio)	≥ 0.25– ≤ 0.5% cetrimonium chloride	SM2	2.3:1	1	0.2 [− 0.3–0.6]	Yes (3/3)
				5	0.4 [− 0.1–0.8]	Yes (3/3)
				10	0.2 [− 0.2–0.6]	Yes (3/3)
Panbio COVID-19 Ag rapid test device buffer (Abbott)	0.49% Tween-20	PDRR	3:1	1	0.2 [− 0.3–0.6]	Yes (3/3)
				5	0.6 [0.1–1.0]	Yes (3/3)
				10	0.2 [− 0.2–0.7]	Yes (3/3)
Healgen coronavirus Ag rapid test cassette swab buffer (Zhejiang Orient Gene)	Not known	PDRR	3:1	1	0.8 [0.4–1.2]	Yes (3/3)
				5	1.4 [1.0–1.8]	Yes (3/3)
				10	1.6 [1.2–2.0]	Yes (3/3)
LumiraDx SARS-CoV-2 Ag test extraction buffer (LumiraDx)	0.5% unspecified detergent 1.00% Tween 20	PDRR	7.5:1	1	− 0.4 [− 0.9–0.0]	Yes (3/3)
				5	− 0.5 [− 0.9– − 0.1]	Yes (3/3)
				10	− 0.5 [− 1.0–0.0]	Yes (3/3)
COVID-19 (SARS-CoV-2) antigen test kit extraction reagent (Anhui Deepblue Medical Technology)	Not known	None	3:1	1	0.0 [− 0.4–0.3]	Yes (3/3)
				5	0.0 [− 0.6–03]	Yes (3/3)
				10	0.1 [− 0.3–0.5]	Yes (3/3)
Veritor extraction reagent (BD)	0.1– < 1% Triton X-100	PDRR	3.25:1	1	≥ 4.5 [4.2–4.8]	No^3^
				5	≥ 5.4 [5.2–5.7]	No^2^
				10	≥ 5.7 [5.4–6.0]	No^1^
SARS-CoV-2 quick antigen extraction buffer (ScheBo)	< 1% Triton X-100	PDRR	2.5:1	1	5.3 [5.1–5.6]	Yes (3/3)
				5	≥ 6.0 [5.7–6.3]	Yes (2/3)
				10	≥ 6.0 [5.7–6.3]	No^4^
Vstrip COVID-19 antigen rapid test extraction buffer (Panion & BF Biotech)	< 1% Tergitol < 1.2% Methanol	PDRR	10:1	1	0.1 [− 0.3–0.4]	Yes (3/3)
				10	0.0 [− 0.5–0.3]	Yes (3/3)
SARS-CoV-2 antigen qualitative test extraction solution (Innova)	Not known	None	2:1	1	0.1 [− 0.3–0.5]	Yes (3/3)
				5	0.1 [− 0.2–0.5]	Yes (3/3)
				10	0.0 [− 0.5–0.4]	Yes (3/3)
COVID-19 rapid antigen test sample buffer (Mologic)	0.4% unspecified surfactant	PDRR	3.5:1	1	≥ 5.4 [5.1–5.7]	No^1^
Extraction buffer from Standard Q COVID-19 Ag Test Kit (SD Biosensor)	Not known	PDRR	2:1	1	1.7 [1.3–2.1]	Yes (3/3)
				5	2.4 [2.0–2.8]	Yes (3/3)
				10	2.9 [2.4–3.3]	Yes (3/3)
Extraction buffer from Standard Q COVID-19 Ag Saliva test kit (SD biosensor)	Not known	PDRR	3.5:1	1	0.8 [0.4–1.2]	Yes (3/3)
				5	1.1 [0.7–1.6]	Yes (3/3)
				10	1.8 [1.4–2.2]	Yes (3/3)

*As indicated on product safety data sheets or other product literature.

^†^Values are given as ≥ when at least one replicate was below the limit of detection.

^‡^PDRR: Pierce Detergent Removal Resin; SM2: Biorad SM2 BioBeads; Limit of detection: ^1^1.0 log_10_ TCID_50_/mL; ^2^1.3 log_10_ TCID_50_/mL; ^3^2.2 log_10_ TCID_50_/mL; ^4^0.7 log_10_ TCID_50_/mL; ^5^1.7 log_10_ TCID_50_/mL.

## Discussion

Incorporation of novel products into existing diagnostic workflows requires them to be effective at inactivating pathogens; this study has provided inactivation efficacy data for twenty-four new products against SARS-CoV-2. Many new nucleic acid extraction reagents are guanidine-free and their inclusion in diagnostic procedures in place of hazardous guanidine-based reagents has the potential to eliminate the chemical hazards associated with guanidine use. Furthermore, many new products are supplied as non-hazardous: non-hazardous inactivating specimen transport media have the potential to increase the safety of sample transport and the speed of testing processes while not posing a chemical hazard to users at the sampling stage. SARS-CoV-2 inactivation effectiveness of twelve buffers provided with LFIA tests was additionally evaluated in this study, since these are being increasingly employed as COVID-19 testing capabilities are scaled up to include home and workplace testing.

Of the products that were demonstrated to inactivate SARS-CoV-2 effectively (i.e. > 4 log_10_ reduction in titre following treatment), four nucleic acid extraction buffers (MELT medium B, D-Biotech Virus Preservation Solution, Abbott ID NOW Elution Buffer, PROmate Sample Preparation Buffer, and two LFIA buffers (BD Veritor and Schebo) are known to contain the non-denaturing detergent Triton X-100 as an active ingredient; we and others have previously shown that the Triton X-100 is effective at reducing SARS-CoV-2 titres by > 5–6 log_10_^6,10^. Commercial products containing Triton X-100 reduced (PROmate and MELT V3), Triton CG-110 (MELT V4), SDS (OMNIgene Oral), trifluoroacetamide/trimethylglycine (virusPHIX+, -LV and -P9), sodium hydroxide (VitaPCR), sodium lauroyl sarcosinate (Zeesan Saliva RNA Collector Kit buffer) were also effective at reducing SARS-CoV-2 infectivity. Several guanidine-free products (Geneme FRANKD and SAVD buffers, GeneFix and BuccalFix) and one LFIA buffer (Mologic) were effective but information on chemical composition for these buffers has not been disclosed by the manufacturers. Enzymatic inactivation may be an alternative to chemical inactivation, and the inactivation of viruses has been previously demonstrated using proteolytic enzymes^11^. Virucidal activity of the enzyme-based inactivants Salicovgel-1 and Salicovgel-2 were assessed in this study; while a > 4 log10 titre reduction could not be demonstrated by either of these products, Salicovgel-2 reduced SARS-CoV-2 titre by ≥ 3 log_10_ after 1-h treatment, indicating potential for enzymatic products as SARS-CoV-2 inactivants. Data presented in this study will aid testing laboratories that are required to replace Triton X-100 with Triton X-100 alternatives, due to the inclusion of Triton X-100 on the European Authorisation list (Annex XIV) of the Registration, Evaluation, Authorisation and Restrictions of Chemicals (REACH) for phasing out of use^12,13^. In addition, several of the products shown to be effective inactivators of SARS-CoV-2 are marketed as non-hazardous specimen transport media, indicating that these may have potential for use in home-sampling kits.

LFIA tests require mixing of a test swab or specimen with the buffer followed by use of a pipette or dropper to load a test cassette; the LFIA testing procedure may therefore lead to the generation of infectious aerosols. Furthermore, the infectious titre of samples tested directly after patient sampling (as is the case for POC tests) is likely to be higher than for those that are transported from sampling sites to testing laboratories before further handling. Inactivation efficacy of POC test buffers is a key consideration for risk assessment of POC testing processes: only three of the twelve LFIA buffers tested in this study were effective at inactivating SARS-CoV-2. Several LFIA buffers tested were completely ineffective against SARS-CoV-2 and of those that did reduce virus titre, most gave modest reductions in comparison with other extraction buffers. Data presented here indicate that testing centres should not rely upon LFIA buffers to completely inactivate infectious samples and that additional control measures should be implemented to ensure the protection of test operators.

Data on the effectiveness of any inactivation step is crucial for designing and risk assessing testing procedures. Findings presented here are relevant for SARS-CoV-2 diagnostic laboratories, testing centres and mass testing programmes worldwide, providing data to support evidence-based risk assessment of testing procedures.
